# Development of *D*-Limonene Nanoemulsions for Oral Cancer Inhibition: Investigating the Role of Ostwald Ripening Inhibitors and Cell Death Mechanisms

**DOI:** 10.3390/ijms26115279

**Published:** 2025-05-30

**Authors:** Suwisit Manmuan, Yotsanan Weerapol, Tiraniti Chuenbarn, Sontaya Limmatvapirat, Chutima Limmatvapirat, Sukannika Tubtimsri

**Affiliations:** 1 Faculty of Pharmaceutical Sciences, Burapha University, Chon Buri 20131, Thailand; suwisit@go.buu.ac.th (S.M.); yotsanan@go.buu.ac.th (Y.W.); tiraniti.ch@go.buu.ac.th (T.C.); 2 Department of Industrial Pharmacy, Faculty of Pharmacy, Silpakorn University, Nakhon Pathom 73000, Thailand; limmatvapirat_s@su.ac.th (S.L.); limmatvapirat_c@su.ac.th (C.L.); 3 Natural Products Research Center, Faculty of Pharmacy, Silpakorn University, Nakhon Pathom 73000, Thailand

**Keywords:** *D*-limonene, nanoemulsion, Ostwald ripening, oral cancer, cell death mechanism

## Abstract

The aim of this study was to investigate the effect of Ostwald ripening inhibitors on *D*-limonene (*D*-LMN) nanoemulsions and to elucidate their impact on oral cancer cells. Various inhibitors, including olive oil, soybean oil, and perilla oil, were incorporated into *D*-LMN nanoemulsions at different ratios (25:75–75:25, *D*-LMN to inhibitor). The resulting nanoemulsions were evaluated for droplet size, size distribution, zeta potential, stability, droplet morphology, cytotoxicity, antimetastatic and anti-invasive activities, apoptosis induction, and cell cycle arrest. Results showed that the 75:25 *D*-LMN to inhibitor ratio produced the smallest droplet size and exhibited great stability, particularly with perilla oil. Notably, *D*-LMN nanoemulsions displayed strong anti-oral cancer effects by reducing cell viability, metastasis, and invasion. Apoptosis was induced, as evidenced by nuclear fragmentation, Annexin V binding, and altered expression of *BAX*, *BCL-XL*, *Cytochrome c*, and *Caspase-9*. Additionally, the nanoemulsions caused cell cycle arrest via downregulation of *Cyclin D1*, *CDK2*, *CDK4*, and *CDK6*. These findings highlight the potential of *D*-LMN nanoemulsions as a promising alternative therapeutic strategy for oral cancer treatment.

## 1. Introduction

Oral cancer, a common form of head and neck cancer, shows a rising annual incidence and remains associated with high mortality rates, particularly in developing countries [1,2]. It typically originates in the oral cavity and can spread to adjacent tissues and distant organs. Early symptoms often resemble benign conditions such as canker sores, resulting in delayed diagnosis and treatment. Management of oral cancer depends on its stage and commonly involves surgery, radiation, and chemotherapy, often used in combination to improve therapeutic efficacy [3]. However, conventional treatments, especially chemotherapy, are associated with significant side effects that negatively impact patient’s quality of life. As a result, alternative therapeutic strategies, including the use of natural compounds, are gaining increasing attention for reducing side effects and enhancing treatment outcomes.

Limonene, a chiral terpene compound, exists as two enantiomers: *D*-limonene (*D*-LMN) and *L*-limonene [4]. *D*-LMN has attracted considerable interest due to its pharmacological properties, especially its anticancer potential. Several studies have demonstrated that *D*-LMN effectively inhibits various cancer cell types, including those of the lung [5], breast [6], and colorectal [7]. However, research on its efficacy and underlying mechanisms against oral cancer remains limited. Despite its strong anticancer potential, the clinical application of *D*-LMN is hindered by its poor water solubility, which limits its bioavailability and therapeutic effectiveness. To overcome these challenges, water-miscible nano-drug delivery systems have emerged as a promising solution.

Nanoemulsions, particularly oil-in-water (O/W) systems, have gained considerable attention due to their improved stability [8], high bioavailability [9], and ability to encapsulate lipophilic compounds and essential oils [10,11]. Several studies have demonstrated the successful use of nanoemulsions for delivering essential oils to inhibit cancer cells. For instance, Jawaid et al. demonstrated that nanoemulsions enhanced the anticancer activity of citronella oil [12], while Manaa et al. reported increased stability and anticancer effects of oregano oil using a nanoemulsion system [13]. Similarly, Alharbi et al. developed a ginger oil nanoemulsion that improved oral absorption and boosted anticancer efficacy [14]. In our previous study, nanoemulsions significantly enhanced the anti-oral cancer activity of spearmint oil compared to its conventional solution form [15]. Given these advantages, nanoemulsions are considered promising delivery systems for *D*-LMN. However, a key challenge in formulating essential oil nanoemulsions is Ostwald ripening, a process in which droplet size increases over time, leading to instability [16]. This phenomenon can be mitigated by incorporating Ostwald ripening inhibitors, which decrease the solubility of the dispersed phase and increase the droplet’s entropy, thereby enhancing emulsion stability. Previous studies have identified several effective inhibitors, such as virgin coconut oil (VCO) [15], olive oil (OLO) [17], soybean oil (SBO) [15], perilla oil (PRO) [15], corn oil [18], and medium-chain triglycerides [19]. Although the role of these inhibitors has been studied, the specific impact of Ostwald ripening inhibitors in *D*-LMN nanoemulsions remains unexplored—particularly due to differences in the chemical structures and properties of various essential oils.

The objective of this research was to investigate the effects of fixed oil type (triglyceride-rich oils) and concentration on the formation and stability of *D*-LMN nanoemulsions, specifically focusing on their role as Ostwald ripening inhibitors. The fixed oils used in this study comprised a diverse range of triglyceride structures, including OLO containing oleic acid, SBO containing linoleic acid, and PRO-containing linolenic acid. Additionally, this research aimed to elucidate the mechanism of cell death induced by *D*-LMN nanoemulsions, thereby contributing to a deeper understanding of their therapeutic potential against oral cancer cells.

## 2. Results

### 2.1. Droplet Size, Size Distribution (Polydispersity Index; PDI) and Zeta Potential

**Figure 1:**
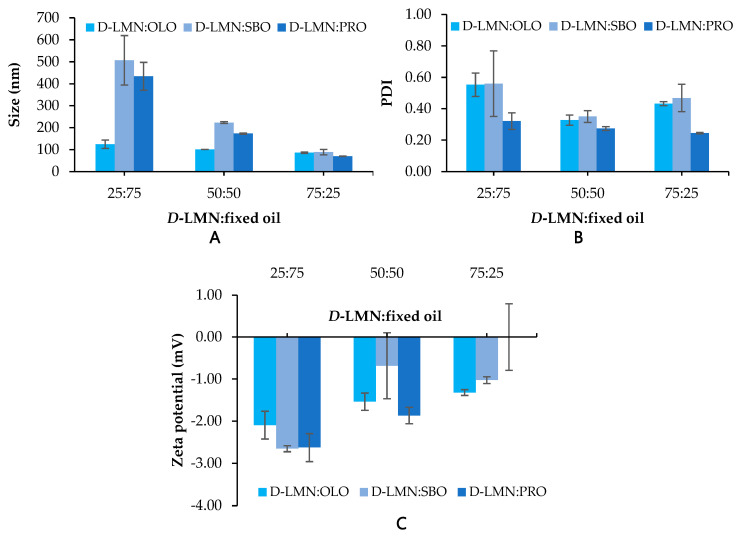
Effect of the fixed oil type and *D*-limonene (*D*-LMN): fixed oil ratio on droplet size (**A**), polydispersity index (PDI; **B**), and zeta potential (**C**) (*n* = 3).

The droplet size and PDI of nanoemulsions prepared using different types of fixed oils and varying ratios of *D*-LMN to fixed oil are presented in Figure 1A,B. Among the various ratios tested, the 75:25 ratio of *D*-LMN to OLO, SBO, and PRO-resulted in the smallest droplet sizes—86.03 ± 3.08 nm, 88.60 ± 12.40 nm, and 70.19 ± 1.23 nm, respectively. The 50:50 ratio of *D*-LMN to OLO produced a droplet size of approximately 100 nm, while all other ratios exhibited droplet sizes exceeding 100 nm. The PDI values for both the 50:50 and 75:25 were less than 0.5.

As shown in Figure 1C, the zeta potential of the *D*-LMN nanoemulsions ranged from –2.66 to 0 mV, with formulations containing a higher proportion of fixed oil exhibiting more negative values. Notably, the 75:25 ratio yielded zeta potentials closest to zero, with the PRO-based nanoemulsion showing the nearest value to neutrality (0.00 ± 0.79 mV).

**Figure 2:**
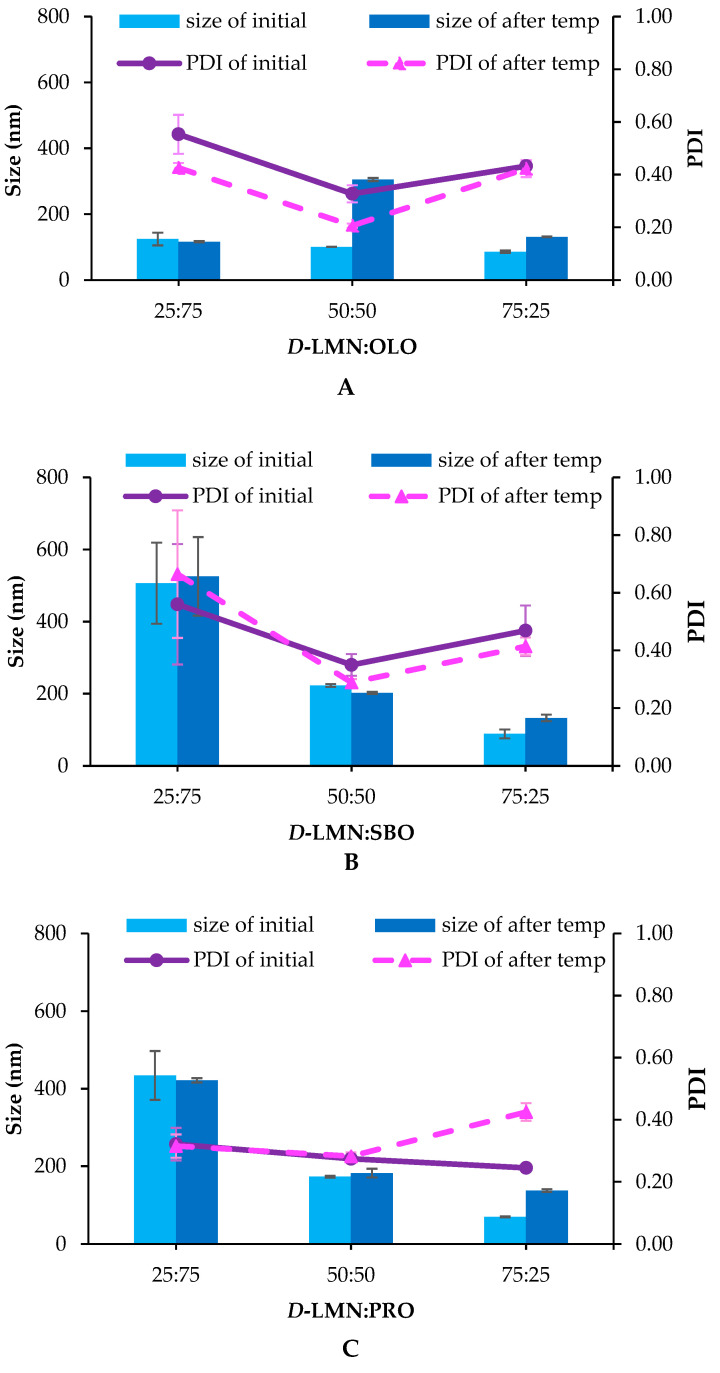
Droplet size and polydispersity index (PDI) of *D*-limonene (*D*-LMN):OLO (**A**), *D*-LMN:SBO (**B**) and *D*-LMN:PRO (**C**) at the initial time point and after the temperature cycling test (*n* = 3).

After undergoing the temperature cycling test, the droplet size of the 75:25 nanoemulsion increased to approximately 130 nm, while the PDI remained below 0.5, indicating acceptable stability. The 50:50 ratio showed the smallest variation in droplet size before and after the temperature cycling test, except in the case of OLO (Figure 2). Other ratios exhibited more noticeable deviations from their initial droplet sizes.

Among the tested formulations, PRO was able to form nanoemulsions with small droplet sizes and narrow size distributions, particularly at the 75:25 ratio. Furthermore, as reported by Muranush et al. [20], PRO has the potential to enhance the activity of active compounds. This may be attributed to the ability of fatty acids with lower melting points to increase cancer cell membrane permeability, thereby promoting the anticancer activity of encapsulated substances. Of the fixed oils evaluated, PRO-exhibited the lowest melting point (−11 °C), compared to SBO (−5 °C) and OLO (8–10 °C). Given its favorable physical properties and its potential to enhance the efficacy of *D*-LMN, PRO was selected as the Ostwald ripening inhibitor for further analysis in *D*-LMN nanoemulsion.

### 2.2. Morphological of Oil Droplet

**Figure 3:**
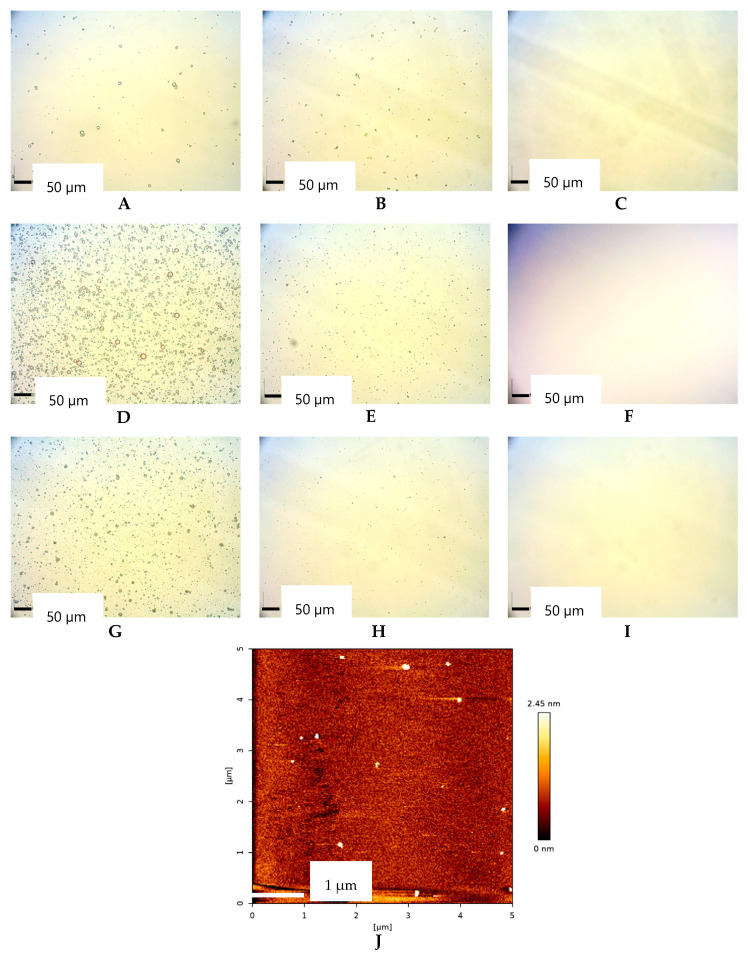
Optical microscopy images of nanoemulsions prepared using different fixed oil types and *D*-limonene (*D*-LMN):fixed oil ratios: *D*-LMN:OLO at 25:75 (**A**), 50:50 (**B**), 75:25 (**C**); *D*-LMN:SBO at 25:75 (**D**), 50:50 (**E**), 75:25 (**F**); *D*-LMN:PRO at 25:75 (**G**), 50:50 (**H**), 75:25 (**I**); and AFM image of *D*-LMN:PRO at 75:25 (**J**).

Figure 3 illustrates the morphological characteristics of oil droplets in *D*-LMN nanoemulsions. Spherical droplets were observed in the 25:75 ratio, while smaller spherical droplets were noted in the 50:50 ratio. However, droplets in the 75:25 ratio could not be visualized using an optical microscope due to their size falling below the detection limit. Therefore, the 75:25 *D*-LMN to PRO-formulation was selected as a representative nanoemulsion for droplet morphology analysis using atomic force microscopy (AFM). The AFM results confirmed that the droplets were spherical and exhibited no signs of aggregation.

### 2.3. Anticancer Activity

**Figure 4:**
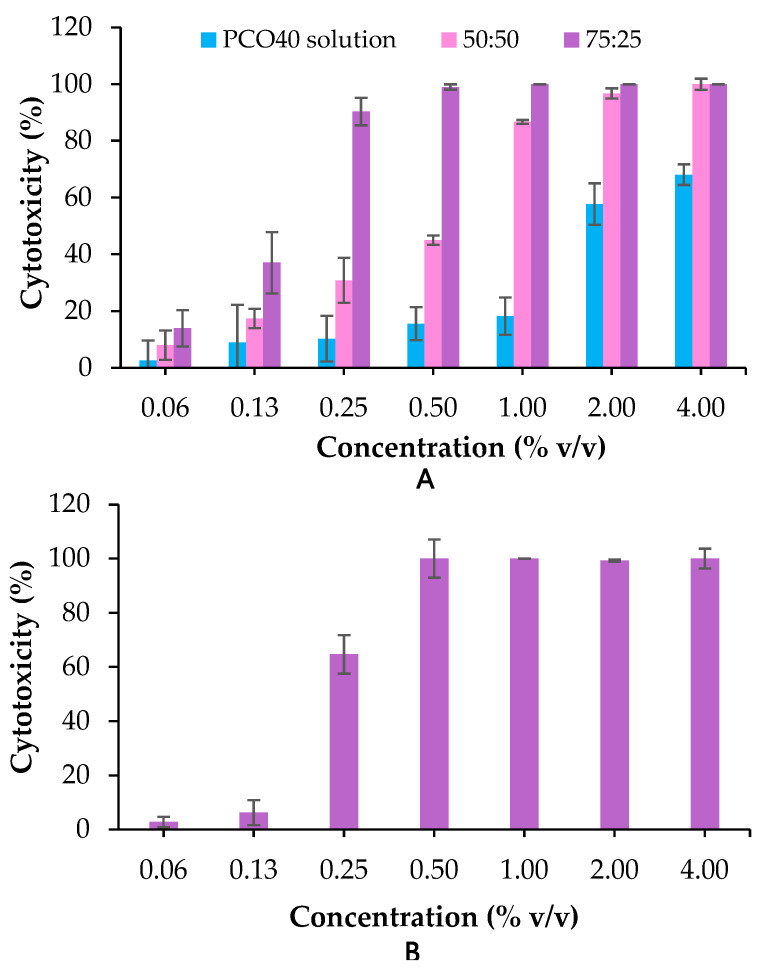
Cytotoxicity following treatment with *D*-limonene (*D*-LMN):PRO-nanoemulsions at 50:50 and 75:25 ratios and PCO40 solution on KON cells (**A**) and *D*-LMN:PRO (75:25) nanoemulsion on MRC-5 cells (**B**) (*n* = 3).

The anticancer activity of *D*-LMN nanoemulsions was evaluated using the MTT assay. The 50:50 and 75:25 *D*-LMN to PRO-ratios were chosen to assess the effect of concentration on cytotoxicity. As Cremophor RH40 (PCO40), a nonionic surfactant, is known to induce cancer cell death [21], a PCO40 solution at an equivalent concentration was included in the experiment. As shown in Figure 4A, the 75:25 nanoemulsion exhibited significantly higher cytotoxicity compared to the 50:50 formulation at all tested concentrations. Complete inhibition of oral cancer (KON) cell viability was observed at concentrations above 0.5% *v*/*v* for the 75:25 ratio, whereas the 50:50 ratio achieved similar efficacy only at concentrations above 2% *v*/*v*. PCO40 solution showed limited cytotoxicity, reducing oral cancer cell viability by approximately 50% only at concentrations exceeding 2% *v*/*v*. Additionally, the 75:25 nanoemulsion was tested on normal fibroblast cells (MRC-5), as shown in Figure 4B. At concentrations above 0.5% *v*/*v*, the nanoemulsion exhibited high cytotoxicity, with more than 99% cell death. However, at concentrations below 0.25% *v*/*v*, it showed reduced cytotoxicity toward normal cells compared to its effects on cancer cells.

The IC_50_ and IC_80_ values of the 50:50 *D*-LMN to PRO-nanoemulsion against oral cancer cells were 0.42% ± 0.14% *v*/*v* and 0.83% ± 0.34% *v*/*v*, respectively. The 75:25 formulation exhibited greater potency, with IC_50_ and IC_80_ values of 0.16% ± 0.02% *v*/*v* and 0.24% ± 0.03% *v*/*v*, respectively. Given its superior efficacy, the 75:25 *D*-LMN nanoemulsion was selected for subsequent experiments.

### 2.4. Antimetastatic and Anti-Invasive Properties

**Figure 5:**
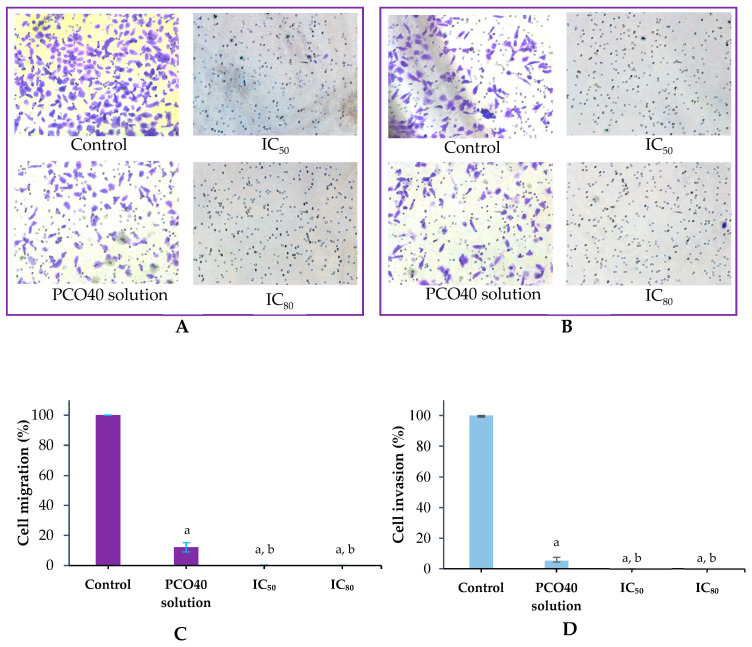
Migration (**A**) and invasion (**B**) of KON cells; Percentage of cell migration (**C**); Percentage of cell invasion (**D**) following treatment with *D*-limonene (*D*-LMN):PRO-nanoemulsions 75:25 ratios at IC_50_, IC_80_ and PCO40 solution at a concentration equivalent to IC_80_. a indicates a significant difference compared to the control, and b indicates a significant difference compared to the PCO40 solution (*p* < 0.05) (*n* = 3).

To evaluate its antimetastatic and anti-invasive properties, the 75:25 *D*-LMN to PRO-nanoemulsion was tested at both IC_50_ and IC_80_ concentrations to assess dose-dependent effects. As shown in Figure 5A,C, both concentrations significantly reduced cell metastasis compared to the control and PCO40 solution-treated groups. The cell migration percentages at IC_50_ and IC_80_ were 0.12% ± 0.13% and 0.04% ± 0.09%, respectively, while the PCO40 solution group exhibited a migration percentage of 12.15% ± 3.10%.

Similarly, the anti-invasion assay results, presented in Figure 5B,D, demonstrated a substantial decrease in cell invasion for the nanoemulsion-treated groups. The invasion percentages for the PCO40 solution groups were 5.45% ± 2.22%, whereas IC_50_ and IC_80_ showed invasion rates of 0.04% ± 0.09% and 0%, respectively.

### 2.5. Apoptosis Induction

**Figure 6:**
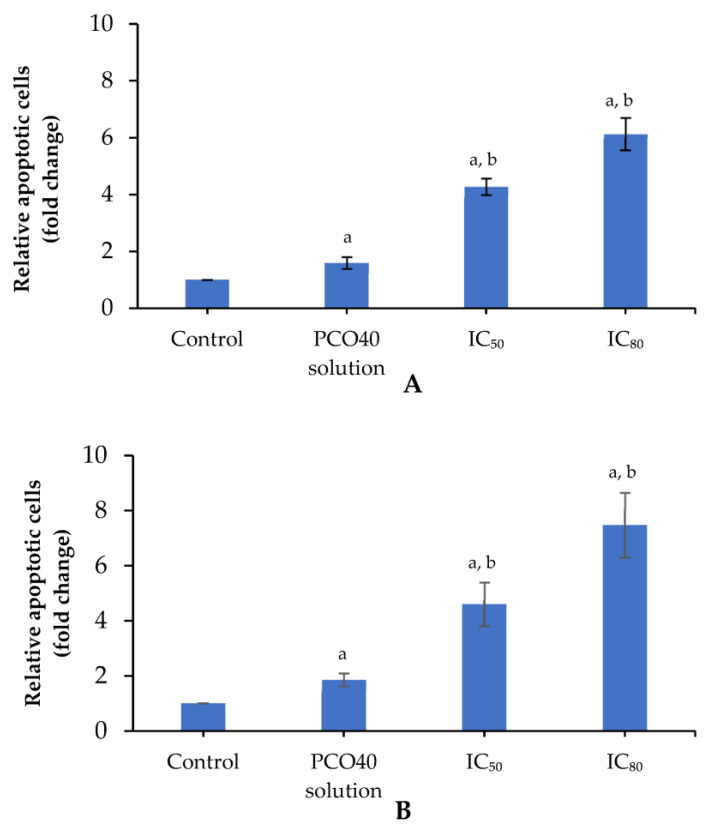
Relative apoptotic cell levels following treatment with IC_50_ and IC_80_ concentrations of *D*-limonene (*D*-LMN):PRO (75:25) nanoemulsion and PCO40 solution at a concentration equivalent to IC_80_, as identified by Hoechst 33258 staining (**A**) and flow cytometry (**B**). a indicates a significant difference compared to the control, and b indicates a significant difference compared to the PCO40 solution (*p* < 0.05) (*n* = 3).

Apoptosis in cancer cells can be assessed using various methods, including nuclear fragmentation analysis and Annexin V staining detected by flow cytometry, which allow for the identification and quantification of apoptotic cells. To examine the dose-dependent apoptotic effect, the 75:25 *D*-LMN:PRO-nanoemulsion was evaluated at both IC_50_ and IC_80_ concentrations. Figure 6A shows the relative levels of apoptotic cells assessed by Hoechst 33258 nuclear staining. Compared to the control and PCO40 solution-treated groups, the nanoemulsion-treated cells exhibited a significant increase in apoptosis. Specifically, the IC_50_ and IC_80_ treatments resulted in 4.28 ± 0.29-fold and 6.13 ± 0.57-fold increases in apoptotic cell count, respectively, relative to the control group. The PCO40 solution group showed a comparatively lower increase in apoptotic cells, with a 1.60 ± 0.21-fold change.

Figure 6B presents the apoptotic cell count by flow cytometry. The relative apoptotic cell counts at IC_50_ and IC_80_ of the 75:25 *D*-LMN:PRO-nanoemulsion were 4.60 ± 0.79-fold and 7.47 ± 1.18-fold compared to control, respectively. PCO40 solution exhibited a slight inhibition of apoptosis induction, with a value of 1.86 ± 0.24-fold compared to the control group.

### 2.6. Cell Cycle Arrest

**Figure 7:**
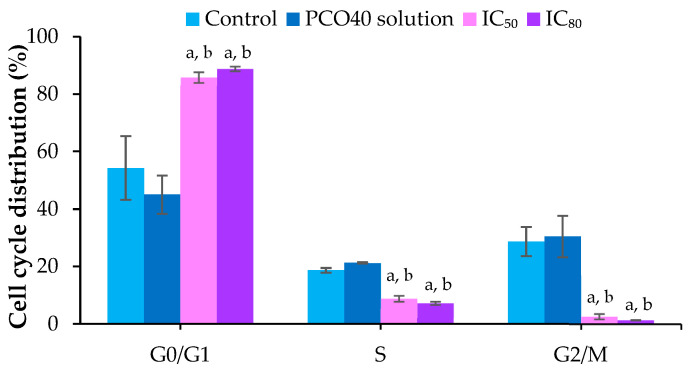
Cell cycle distribution in KON cells after treatment with *D*-limonene (*D*-LMN):PRO (75:25) nanoemulsion at IC_50_ and IC_80_ concentrations_,_ and PCO40 solution at a concentration equivalent to IC_80_. a indicates a significant difference compared to the control, and b indicates a significant difference compared to the PCO40 solution (*p* < 0.05) (*n* = 3).

To enhance understanding of the effects of *D*-LMN nanoemulsion on the cell cycle, flow cytometry with propidium iodide (PI) staining was employed. The 75:25 *D*-LMN:PRO-nanoemulsion was tested at IC_50_ and IC_80_ to evaluate its dose-dependent effects. Figure 7 presents the cell cycle distribution in the G0/G1, S, and G2/M phases after treatment with the nanoemulsion at IC_50_ and IC_80_ and with PCO40 solution, compared to the control. The percentage of G0/G1 cells was higher at IC_50_ and IC_80,_ while the number of cells in the G2/M phase was distinctly reduced compared to the PCO40 solution and the control group. The IC_50_ and IC_80_ of the *D*-LMN nanoemulsion inhibited the accumulation of cells in the S phase compared to the PCO40 solution and the control group.

### 2.7. Gene Expression

**Figure 8:**
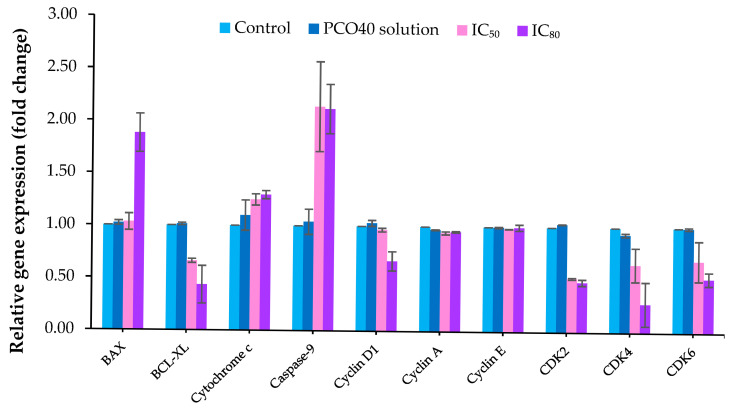
Gene expression levels after treatment with *D*-limonene (*D*-LMN):PRO (75:25) nanoemulsion at IC_50_ and IC_80_ concentrations and PCO40 solution at a concentration equivalent to IC_80_, as assessed by RT-PCR (*n* = 3).

Reverse transcription polymerase chain reaction (RT-PCR) was also employed to analyze the expression of apoptosis-related genes, including *BAX*, *BCL-XL*, *Cytochrome c*, and *Caspase-9*, to determine the type of cell death. In this experiment, the 75:25 *D*-LMN:PRO at both IC_50_ and IC_80_ was studied to verify the dose-dependent effect. At IC_80_, *D*-LMN nanoemulsion altered gene expression, with fold changes of 1.88 ± 0.19, 0.43 ± 0.18, 1.29 ± 0.04, and 2.12 ± 0.24 for *BAX*, *BCL-XL*, *Cytochrome c*, and *Caspase 9*, respectively, as shown in Figure 8. At IC_50_, the expression levels of *BCL-XL*, *Cytochrome c*, and *Caspase-9* were also modulated, with fold changes of 0.66 ± 0.02, 1.25 ± 0.05, and 2.14 ± 0.43, respectively, compared to the control group. However, *BAX* expression was not affected by the *D*-LMN nanoemulsion at IC_50_. The PCO40 solution did not affect the expression of apoptosis-related genes.

Furthermore, RT-PCR was conducted to analyze the expression levels of genes related to cell cycle arrest, including *Cyclin D1*, *Cyclin A*, *Cyclin E*, *CDK2*, *CDK4*, and *CDK6*. The 75:25 ratio of *D*-LMN to PRO at IC_50_ and IC_80_ was employed for this test to verify the dose-dependent effect. Figure 8 demonstrates the alterations in *Cyclin* and *CDK* gene expression after induction with the *D*-LMN nanoemulsion. The results showed that the *D*-LMN nanoemulsion at IC_80_ altered the expression of *Cyclin D1*, *CDK2*, *CDK4*, and *CDK6*, with fold changes of 0.67 ± 0.09, 0.48 ± 0.03, 0.27 ± 0.21, and 0.52 ± 0.11, respectively. The *D*-LMN nanoemulsion at IC_50_ exhibited similar activity, with fold changes of *CDK2*, *CDK4*, and *CDK6* showing 0.51 ± 0.01 times, 0.65 ± 0.16 times, and 0.69 ± 0.19 times, respectively, compared to the control group. Notably, *D*-LMN did not affect the expression of *Cyclin A* and *Cyclin E*. A slight decrease in *CDK4* expression was observed following treatment with the PCO40 solution.

## 3. Discussion

Droplet size and size distribution significantly influence the properties and effectiveness of nanoemulsions. Evaluating them is essential to ensure optimal performance and long-term stability of nanoemulsions. In this study, various types and concentrations of fixed oils were investigated. Among the different ratios tested, the 75:25 formulation exhibited the smallest droplet sizes. The PDI values for both 50:50 and 75:25 were below 0.5, indicating a narrow size distribution [22]. At the 75:25 ratio, PRO-yielded the smallest droplet size and the narrowest size distribution. Observations under the microscope were consistent with results from the size analyzer. Furthermore, the discrete spherical shape found in the 75:25 *D*-LMN to PRO-ratio indicated good physical stability against Ostwald ripening [23]. The zeta potential at the 75:25 ratio was closest to zero compared to other formulations, suggesting complete coverage by the nonionic surfactant [15]. These findings suggest that both the type and ratio of fixed oil influence the droplet size and size distribution of *D*-LMN nanoemulsions. It appears that the short-bent triglyceride structure—particularly the linolenic acid component in PRO, with an approximate chain length of 35.7 Å [15]—is more favorable than the longer-bent structures of oleic acid (OLO: 35.9 Å) and linoleic acid (SBO: 36.3 Å) [15] for nanoemulsion formation. Droplet size may be affected by the hydrophilic-hydrophobic balance and hydrogen bonding interaction among *D*-LMN, PCO40, and triglycerides [15,17,24]. Consequently, differences in oil type and ratio lead to distinct droplet formation, resulting in variations in droplet size and size distribution.

In addition to their role in the formation of *D*-LMN nanoemulsions, the influence of Ostwald ripening inhibitors on physical stability was also investigated. The nanoemulsions were subjected to a temperature cycling test as an accelerated method to assess the effect of these inhibitors on emulsion stability. After the test, changes in droplet size were observed, likely due to Ostwald ripening [25]. This thermodynamically driven process plays a critical role in the stability of essential oil nanoemulsions. It occurs as smaller droplets, with higher surface energy, dissolve and redeposit onto larger droplets to minimize interfacial energy. The solubility of oil in the continuous phase is the primary factor influencing the rate of Ostwald ripening, as described by Liftshitz and Slyozov and Wagner (LSW theory) [26]. Due to its relatively high water solubility, *D*-LMN tends to accelerate the Ostwald ripening process. Fixed oils, possessing larger molar volumes and lower water solubility, offer greater resistance to Ostwald ripening [27]. Their presence increases entropy within the droplet system, thereby providing a thermodynamic barrier that suppresses the ripening process [27,28]. The concentration of fixed oil directly impacted the Ostwald ripening rate, as observed in the 50:50 and 75:25 formulations. However, using a high concentration of fixed oil may lead to larger droplet sizes [24], which could potentially reduce their permeability into cancer cells. These results highlight the importance of selecting an appropriate ratio between essential oil and fixed oil in nanoemulsion preparation, which is critical for maintaining emulsion stability.

Regarding anti-oral cancer activities, *D*-LMN nanoemulsions exhibited inhibitory effects on oral cancer cells in a concentration-dependent manner. These findings are consistent with those of Yu et al., who reported that *D*-LMN showed dose-dependent inhibitory effects on lung cancer proliferation [5]. The low activity of PCO40 is attributed to its surfactant properties, which can compromise the barrier function and induce lysis of cancer cell membranes [29]. The results of cytotoxicity on normal fibroblasts also suggested that *D*-LMN demonstrates greater selectivity toward cancer cells at concentrations below 0.25% *v*/*v*. Nonetheless, further studies are necessary to assess its toxicity in in vivo models.

Metastasis and invasion contribute to cancer lethality by enabling the spread to distant organs and infiltration into tissues, thereby complicating treatment and disease control [30,31]. *D*-LMN exhibited potent antimetastatic and anti-invasive activities against oral cancer cells in a concentration-dependent manner. Consistent with previous findings, *D*-LMN has been shown to suppress peritoneal and hepatic metastasis in gastric cancer [32] and to inhibit metastasis in colon cancer cells [33].

Apoptosis is a programmed form of cell death that occurs without triggering inflammation or causing tissue damage, making it an attractive target for anticancer therapy [34,35]. Hallmark features of apoptosis include nuclear fragmentation, externalization of phosphatidylserine on the outer cell membrane, and altered expression of apoptotic genes such as *BAX*, *BCL-XL*, *Cytochrome c*, and *Caspase-9*. In our experiment, apoptotic characteristics were examined using fluorescent nuclear staining under a microscope, Annexin V-FITC binding to phosphatidylserine detected by flow cytometry, and gene expression analysis via RT-PCR. The results indicated that *D*-LMN nanoemulsion can induce oral cancer cell death through intrinsic apoptosis (mitochondrial pathway) in a dose-dependent manner. The induction of *BAX* (pro-apoptotic) expression facilitates mitochondrial outer membrane permeabilization, thereby promoting the release of proapoptotic factors such as *Cytochrome c*. Concurrently, suppression of *BCL-XL*, an anti-apoptotic member of the *BCL-2* family, enhances the apoptotic response by relieving its inhibitory effect on *BAX*. This cascade leads to the activation of *Caspase-9*, a key initiator of the intrinsic apoptotic pathway. The sequential activation of downstream caspases ultimately results in programmed cell death. Consistent with previous reports, *D*-LMN has been shown to modulate the expression of apoptosis-related genes, including *p53*, *BAX*, and *BCL-2* in colon cancer cells [7], and *BCL-XL*, *Cytochrome c*, and *pro-caspase 9* in lung cancer cells [5], as well as *BCL-2* and *BAX* in skin tumors [36].

Regulation of the cancer cell cycle plays a crucial role in effective cancer treatment [37,38]. Cancer cells often exhibit defects in cell cycle checkpoints, impairing their ability to undergo programmed cell death. In this study, *D*-LMN nanoemulsion induced cell cycle arrest at the G0/G1 phase in a concentration-dependent manner. This arrest restricted the growth and division of oral cancer cells. Moreover, such inhibition can also initiate apoptosis, a natural mechanism for eliminating damaged or undesirable cells [39,40]. Consistent with a previous report, Zhang et al. demonstrated that *D*-LMN induced G0/G1 cell cycle arrest in gastric carcinoma cells [41]. Several studies have also confirmed the cell cycle arrest activity of *D*-LMN. It has been shown to induce G2/M phase arrest in breast cancer cells [6]. Additionally, *D*-LMN formulated in liposomes exhibited G2/M phase arrest in malignant glioma cells [42]. Cell proliferation is generally regulated by various genes at different stages of the cell cycle [43]. *Cyclin D1*, *CDK2*, *CDK4*, and *CDK6* are key regulators facilitating cell cycle progression. *Cyclin D1* partners with *CDK4* and *CDK6* to drive the transition from the G1 to the S phase [44]. *CDK2*, in conjunction with *Cyclin E*, promotes the G1/S transition and later associates with *Cyclin A* during the S phase to initiate DNA synthesis [45]. The suppression of these genes led to cells accumulate in the G1 phase, as observed in the cell cycle arrest analysis. The findings suggest that *D*-LMN nanoemulsion induces cell cycle arrest by downregulating *Cyclin D1*, *CDK2*, *CDK4*, and *CDK6*. This aligns with previous studies that reported the ability of *D*-LMN to inhibit *Cyclin D1* expression in breast cancer [46].

## 4. Materials and Methods

### 4.1. Materials

*D*-LMN (Lot No. Z6PA14) was purchased from MySkin Recipes, Bangkok, Thailand. PCO40 (Lot No. 30696747G0) and OLO (Lot No. L72039B-28776) were obtained from PC Drug Center, Bangkok, Thailand. SBO (Lot No. FM-OP-008) was purchased from Thai Vegetable Oil, Bangkok, Thailand. PRO was obtained from Doitung Thanyapueach, Chiang Rai, Thailand. A human oral squamous cancer cell line (Lot No. 01262007) was procured from the Japanese Collection of Research Bioresources Cell Bank, Tokyo, Japan.

### 4.2. Nanoemulsion Preparation

The *D*-LMN nanoemulsion was produced by the phase inversion temperature (PIT) method. Nanoemulsions were formulated using different fixed oils, including OLO, SBO, and PRO, with *D*-LMN-to-fixed oil ratios ranging from 25:75 to 75:25. The oil phase consisted of *D*-LMN, a selected fixed oil, and PCO40, with a total concentration of 20% *w*/*w*, while the aqueous phase consisted solely of water. Both phases were heated, followed by homogenization at 3800 rpm for 5 min. The resulting nanoemulsions were evaluated for droplet size, size distribution, zeta potential, stability, droplet morphology, cytotoxicity, antimetastatic and anti-invasive activities, apoptosis induction, and cell cycle arrest. The formulation of *D*-LMN nanoemulsion are presented in Table 1.

### 4.3. Measurement of Droplet Size, Size Distribution and Zeta Potential

The droplet size, PDI, and zeta potential of the *D*-LMN nanoemulsion were measured using a Zetasizer Nano ZS (Malvern Instruments, Worcestershire, UK). The nanoemulsion was transferred into a specialized cuvette for analysis. All measurements were conducted in triplicate, and the results were reported as mean ± standard deviation (SD).

### 4.4. Stability Assessment

Nanoemulsion stability was assessed using a temperature cycling test. Three bottles of the nanoemulsion were alternated between 45 °C for 24 h and 4 °C for 24 h (one cycle), repeated for a total of six cycles. Following the test, droplet size, and size distribution were remeasured using the Zetasizer Nano ZS to evaluate any changes in physical characteristics. The results were also expressed as mean ± SD.

### 4.5. Morphological Analysis of Oil Droplets

The morphology of the oil droplets was examined using CX41 RF optical microscopy (Olympus, Tokyo, Japan) and Nanowizard III AFM (Bruker, Berlin, Germany). For optical microscopy, samples were placed on a glass slide and viewed through a digital eyepiece (ANMO Electronics, Hsinchu, Taiwan). Representative nanoemulsions were further analyzed by AFM. Samples were deposited onto freshly prepared mica disks and cured in a fume hood at ambient temperature. A tapping mode nanoprobe cantilever was used to assess the dried nanoemulsion surface.

### 4.6. Anticancer Activity Study

The MTT assay was used to evaluate the anti-oral cancer activity of the *D*-LMN nanoemulsion. MRC-5 cells were employed as a noncancerous reference to assess cytotoxicity and facilitate comparison with cancer cells. Suspensions of KON and MRC-5 cells (5 × 10^3^–1 × 10⁴ cells/well) were separately seeded into 96-well plates and incubated for 24 h. Following incubation, *D*-LMN nanoemulsion and PCO40 solution at concentrations ranging from 0.06% to 4.00% *v*/*v* were added to the wells. After a further 24 h incubation, the treatment solutions were removed, and 3-(4,5-dimethylthiazol-2-yl)-2,5-diphenyltetrazolium bromide (MTT) solution was added. The plates were then incubated in the dark for 3 h. Subsequently, the MTT solution was replaced with dimethyl sulfoxide to dissolve the formazan crystals formed by viable cells. The resulting purple formazan product was quantified using a FLUOstar Omega microplate reader with Omega software version 6.20 (BMG LABTECH, Ortenberg, Germany) at 570 nm. The percentages of cell viability and cytotoxicity were calculated using Equations (1) and (2) (*n* = 3). IC_50_ and IC_80_ values were determined by plotting cell viability percentages against *D*-LMN nanoemulsion concentrations and analyzing the relationship to identify the concentrations at which 50% and 80% of cell viability were inhibited, respectively.

(1) $$\mathrm{Cell}\;\mathrm{viability}\;(\%)=100\;\times \;\;\frac{Mean\;absorbance\;of\;treated\;cells}{Mean\;absorbance\;of\;untreated\;cells}$$

Cytotoxicity (%) = 100 − Cell viability (%)(2)

### 4.7. Transwell Migration Assay

The antimetastatic property of the *D*-LMN nanoemulsion was evaluated using a transwell migration assay. The assessment was conducted with 8-μm pore size cell culture inserts (Corning, Steuben County, New York, NY, USA). KON cells (1000 cells/well) were cultured in serum-free media. Representative nanoemulsions at IC_50_ and IC_80_, PCO40 solution (at the same concentration as the IC_80_ of the nanoemulsion), and Dulbecco’s Modified Eagle Medium (DMEM) (control; untreated group) were added to the upper chamber. Meanwhile, 500 µL of DMEM supplemented with 10% fetal bovine serum (FBS) was added to the lower compartment. After 48 h of incubation, nonmigrated KON cells in the upper chamber were gently removed using a cotton swab. The remaining cells were then fixed with ice-cold methanol, rinsed with phosphate-bufferred saline (PBS), and stained with 0.5% crystal violet for 15 min. After additional PBS washes, the stained migrated cells were counted under an inverted microscope. The percentage of migrated cells was calculated using Equation (3) (*n* = 3).

(3) $$\mathrm{Cell}\;\mathrm{migration}\;(\%)=100\;\times \;\frac{number\;of\;migrated\;cells\;after\;treated\;with\;sample}{number\;of\;migrated\;cells\;of\;untreated\;group}$$

### 4.8. Transwell Invasion Assay

The Matrigel matrix was diluted with serum-free medium and used to coat the upper surface of the invasion chamber with 8-μm pore size (Corning, Steuben County, New York, NY, USA) in a 24-well plate. A total of 1500 KON cells were seeded into each well. Representative nanoemulsions at IC_50_ and IC_80_, PCO40 solution (at the same concentration as in IC_80_) and DMEM (control; untreated group) were introduced into the upper compartment. In the lower compartment, 500 µL of DMEM supplemented with 10% FBS was added. After 48 h of incubation, noninvasive cells were removed from the upper chamber using a cotton swab. Invaded cells were fixed with methanol for 15 min and stained with 0.5% crystal violet for 30 min. The upper compartment was thoroughly washed three times with PBS. The invaded cells were examined using an inverted microscope (Olympus, Tokyo, Japan). The percentage of cell invasion was calculated using Equation (4) (*n* = 3).

(4) $$\mathrm{Cell}\;\mathrm{invasion}\;(\%)=100\;\times \;\frac{number\;of\;invaded\;cells\;after\;treated\;with\;sample}{number\;of\;invaded\;cells\;of\;untreated\;group}$$

### 4.9. Nucleus Analysis by Hoechst 33258 Staining

Nuclear staining with fluorescence was also employed to determine the mode of cell death. After treating the cells (5  ×  10^4^ cells/well) with representative nanoemulsions at IC_50_, IC_80_, PCO40 solution (at the same concentration as in IC_80_), and DMEM (control; untreated group) for 24 h, the cells were washed twice with PBS and fixed with 4% *v*/*v* paraformaldehyde for 10 min. They were then permeabilized with 0.2% *v*/*v* Triton X-100 for 10 min. Following this, the cells were stained with Hoechst 33258 for 10 min in the dark and washed with PBS. The stained nuclei were observed under a blue-filter inverted fluorescence microscope (Nikon, Tokyo, Japan). The relative apoptotic cells was calculated using Equation (5) (*n* = 3).

(5) $$\mathrm{Relative}\;\mathrm{apoptotic}\;\mathrm{cell}\;(\mathrm{fold}\;\mathrm{change})=\frac{number\;of\;apoptotic\;cells\;after\;treated\;with\;sample}{number\;of\;apoptotic\;cells\;of\;untreated\;group}$$

### 4.10. Determination of Cell Death Using Annexin V-FITC/PI Staining with Flow Cytometry

Flow cytometry with Annexin V-FITC/PI staining was employed to determine the type of cell death induced by apoptosis. KON cells (5  ×  10^4^ cells/well) were seeded in 6-well plates and incubated for 24 h. Representative nanoemulsions at IC_50_ and IC_80_, PCO40 solution (tested at a concentration equivalent to the nanoemulsion IC_80_), and DMEM (control; untreated group) were added to the respective wells and incubated for an additional 24 h. Following treatment, cells were harvested using 0.25% Trypsin-EDTA and washed three times with PBS. Prior to flow cytometry analysis, the cells were resuspended in Annexin V binding buffer and stained with 5 µL of Annexin V-FITC and 500 µL of PI; 10 µg/mL) for 15 min in the dark [38]. A total of 10,000 cells per sample were analyzed using a CytoFLEX flow cytometer (Beckman Coulter, Brea, California, United States). Cell populations were categorized as follows: live cells (Annexin−/PI−), early apoptotic (Annexin+/PI−), late apoptotic (Annexin+/PI+), and necrotic (Annexin−/PI+). Apoptosis was defined as the sum of early and late apoptotic cells. The change in apoptotic cell number relative to the control group was calculated using Equation (6) (*n* = 3).

(6) $$\mathrm{Relative}\;\mathrm{apoptotic}\;\mathrm{cell}\;(\mathrm{fold}\;\mathrm{change})=\frac{number\;of\;apoptotic\;cells\;after\;treated\;with\;sample}{number\;of\;apoptotic\;cells\;of\;untreated\;group}$$

### 4.11. Cell Cycle Arrest Analysis

To investigate the impact of *D*-LMN nanoemulsion on cell cycle arrest, the DNA content of KON cells at various stages of the cell cycle was stained with PI. KON cells (5  ×  10^4^ cells/well) were seeded into 6-well plates and incubated for 24 h. Representative nanoemulsions at IC_50,_ and IC_80_ concentrations, PCO40 solution (at the same concentration as in the IC_80_ nanoemulsion), and DMEM (control; untreated group) were added and included for another 24 h. Cells were harvested using 0.25% trypsin-EDTA and washed with cold PBS. The resulting cell pellets were fixed in ice-cold ethanol and subsequently washed twice with PBS. RNase A and PI were then added to the pellets and incubated for 30 min. A total of 1 ×10^4^ cells per sample were analyzed using a CytoFLEX flow cytometer. The distribution of KON cells in the G0/G1, S, and G2/M phases was determined using CytExpert software version 2.4.0.28 (*n* = 3).

### 4.12. Gene Expression Analysis by RT-PCR

The expression of genes related to apoptosis and cell cycle progression was analyzed using RT-PCR. KON cells (5  ×  10^4^ cells/well) were seeded in 6-well plates and incubated for 24 h. Representative nanoemulsions at IC_50_ and IC_80_, PCO40 solution (tested at a concentration equivalent to the nanoemulsion IC_80_), and DMEM (control; untreated) were separately added to the wells. Total RNA was extracted using the GF-1 Total RNA Extraction Kit, and reverse transcription was performed using the ImProm-II™ Reverse Transcription System to synthesize cDNA. PCR amplification was carried out using gene-specific primers and cDNA as a template, with Taq polymerase facilitating the reaction. A FlexCycler2 thermal cycler (Analytik Jena, Jena, Germany) was used with the following conditions: 30 cycles of denaturation at 95 °C for 1 min, annealing for 45 s, extension at 72 °C for 1 min, and a final extension at 72 °C for 10 min. The PCR products were separated via electrophoresis on a 1.5% agarose gel for 20 min [38]. The Alliance Q9 Advanced gel documentation system (UVITEC, Cambridge, UK) was used to quantify the DNA band densities after the gels were stained with VISafe green gel stain. The primer sequences are presented in Table 2.

### 4.13. Statistical Analysis

Statistical analysis was conducted using SPSS version 10.0 for Windows (SPSS Inc., Chicago, IL, USA). One-way ANOVA followed by Tukey’s post hoc test was used to evaluate the data, with significance set at a 95% confidence level.

## 5. Conclusions

This study successfully developed *D*-LMN nanoemulsions using the PIT method by varying the type and concentration of Ostwald ripening inhibitors. The incorporation of fixed oils, particularly at a 75:25 ratio of *D*-LMN to PRO, effectively suppressed Ostwald ripening, resulting in nanoemulsions with optimal droplet size and great physical stability. The *D*-LMN nanoemulsions exhibited notable anticancer activity against oral cancer cells, including inhibition of cell viability, suppression of metastasis and invasion, induction of intrinsic apoptosis, and cell cycle arrest. These findings underscore the potential of *D*-LMN nanoemulsions as a promising natural compound-based strategy for oral cancer treatment, with the added advantage of minimized side effects.

## Figures and Tables

**Table 1:** Formulation of *D*-limonene (*D*-LMN) nanoemulsion.

Ingredient	Concentration (%*w*/*w*)								
	25:75			50:50			75:25		
*D*-LMN	2.5	2.5	2.5	5.0	5.0	5.0	7.5	7.5	7.5
OLO	7.5	-	-	5.0	-	-	2.5	-	-
SBO	-	7.5	-	-	5.0	-	-	2.5	-
PRO	-	-	7.5	-		5.0	-	-	2.5
PCO40	10	10	10	10	10	10	10	10	10
Water	80	80	80	80	80	80	80	80	80

**Table 2:** Primer sequences used for RT-PCR. Primers were designed using Primer-BLAST from the National Center for Biotechnology Information.

Gene	Forward Primers	Reverse Primers
*GAPDH*	5′-AGGGCTGCTTTTAACTCT GGT-3′	5′-CCCCACTTGATTTTGGAGGGA-3′
*BAX*	5′-CCCTTTTGCTTCAGG GTTTC-3′	5′-TGTTACTGT CCA GTT CGT CC-3′
*BCL-XL*	5′-GATCCCCATGGCAGCAGTAAA GCAAG-3′	5′-CCCCATCCCGGAAGAGTTCATTCACT-3′
*Cytochrome c*	5′-CCCAGAAGTACATCCCTGGAAC-3′	5′-GGCAGTGGCCAATTATTACTCA-3′
*Caspase-9*	5′-GCTGTGTCAAGTTTGCCTACCC-3′	5′-CCAGAATGCCATCCAAGGTCTC -3′
*Cyclin D1*	5′-AACTACCTGGACCGCTTCCT-3′	5′- CCACTTGAGCTTGTTCACCA-3′
*Cyclin E*	5′-CGGCCTATATATTGGGTTGGC-3′	5′-GGCTGCTGCTTAGCTTGTAAAC-3′
*Cyclin A*	5′-GCCATTAGTTTACCTGGACCCAGA-3′	5′-CACTGACATGGAAGACAGGAACCT-3′
*CDK2*	5′-GCTTTCTGCCATTCTCATCG -3′	5′-GTCCCCAGAGTCCGAAAGAT-3′
*CDK4*	5′-ACGGGTGTAAGTGCCATCTG-3′	5′-TGGTGTCGGTGCCTATGGGA-3′
*CDK6*	5′-CGAATGCGTGGCGGAGATC -3′	5′-CCACTGAGGTTAGAGCCATC-3′

## Data Availability

Data are available only in this article.
